# Transcriptional and Metabolic Investigation in 5′-Nucleotidase Deficient Cancer Cell Lines

**DOI:** 10.3390/cells10112918

**Published:** 2021-10-28

**Authors:** Octavia Cadassou, Prescillia Forey, Christelle Machon, Edoardo Petrotto, Kamel Chettab, Maria Grazia Tozzi, Jérôme Guitton, Charles Dumontet, Emeline Cros-Perrial, Lars Petter Jordheim

**Affiliations:** 1 Univ Lyon, Université Claude Bernard Lyon 1, INSERM 1052, CNRS 5286, Centre Léon Bérard, Centre de Recherche en Cancérologie de Lyon, 69008 Lyon, France; octavia.cadassou@chu-lyon.fr (O.C.); prescilliaforey@gmail.com (P.F.); christelle.machon@chu-lyon.fr (C.M.); edoardo.petrotto@gmail.com (E.P.); abdelkamel.chettab@univ-lyon1.fr (K.C.); jerome.guitton@chu-lyon.fr (J.G.); charles.dumontet@chu-lyon.fr (C.D.); emeline.perrial@univ-lyon1.fr (E.C.-P.); 2 Hospices Civils de Lyon, Centre Hospitalier Lyon-Sud, 69495 Pierre Bénite, France; 3 Dipartimento di Biologia, Unità di Biochimica, Università di Pisa, Via San Zeno 51, 56127 Pisa, Italy; maria.grazia.tozzi@unipi.it

**Keywords:** CD73, cN-II, metabolic pathways, transcriptional regulation

## Abstract

Enzymes of nucleoside and nucleotide metabolism regulate important cellular processes with potential impacts on nucleotide-unrelated parameters. We have used a set of CRISPR/Cas9-modified cell models expressing both, one, or none of the 5′-nucleotidases cN-II and CD73, together with RNA sequencing and targeted metabolomics, to decipher new regulatory roles of these proteins. We observed important transcriptional modifications between models as well as upon exposure to adenosine. Metabolite content varied differently between cell models in response to adenosine exposure but was rather similar in control conditions. Our original cell models allowed us to identify a new unobvious link between proteins in the nucleotide metabolism and other cellular pathways. Further analyses of our models, including additional experiments, could help us to better understand some of the roles played by these enzymes.

## 1. Introduction

Nucleotide metabolism is a complex cellular process involving an important number of genes, proteins, and enzymes with many redundant functionalities. Nucleotides are involved in a large panel of cellular activities, such as DNA synthesis and repair, transcription, energy homeostasis, and signal transduction. This latter is exemplified by the involvement of nucleotides in the intracellular signal transduction, but also by the purinergic signaling with adenosine- and ATP-receptors present on cell membranes. Modifications in nucleotides and nucleotide metabolism have been shown to be associated with pathologies such as immunologic disorders, rheumatism and gout, cardiovascular diseases, and cancers. This is evidenced by some of these pathologies being associated with protein deficiency or enzymatically inactivating mutations such as immunodeficiency in the case of adenosine deaminase deficiency [1], myopathies for TK2-deficiency [2], hemolytic anemia in *NT5C3A*-mutated patients [3], and a more complex clinical presentation for hCNT1-deficiency [4].

We have a particular interest in the 5′-nucleotidases CD73 (*NT5E*) and cN-II (*NT5C2*) in cancer cells. These two enzymes dephosphorylate purine nucleoside monophosphates into corresponding nucleosides on the extracellular surface (CD73) or in the cytosol (cN-II). Patients with inactivating mutations have been reported for these enzymes as well, with arterial calcifications for CD73-deficiency [5] and hereditary spastic paraplegia for cN-II [6]. CD73-deficient mice have been largely studied since their development [7,8], and cN-II-deficient mice were more recently described [9,10]. We have been interested in these proteins from a therapeutic viewpoint, and developed inhibitors for both CD73 [11,12,13,14] and cN-II [15,16,17] over the last few years. We also demonstrated that cN-II knockdown in cell lines from hematological malignancies was associated with increased sensitivity to nucleoside-based treatments such as 6-mercaptopurine [18]. We are also interested in the biological functions of these proteins in cancer cells, and have developed various models to study this. First, we used siRNA-mediated targeting of cN-II in breast cancer cells to show that their adaptability to glucose deprivation as well as handling of ROS is cN-II-dependent [19]. Further, we developed original CRISPR/Cas9 cells for cN-II and/or CD73 obtaining breast and lung cancer cell models expressing none, both or one of the proteins, thus enabling the evaluation of concomitant roles of the two proteins in cancer cell biology [20,21]. The main findings from our initial work on these cell models are that cN-II is involved in cell migration, most probably through the regulation of Cox-2 expression and prostaglandin E2-production, and that CD73-deficient lung cancer cells had modified sensitivity to certain classical cancer drugs. We also found that the cell models responded differently to exposure to adenosine and AMP.

There is, to our knowledge, no published data on the transcriptomic and metabolomics modifications in human cancer cell lines expressing or not cN-II and/or CD73. There is however, a report on data obtained on siRNA-mediated knock down of cN-II in fruitfly cells [22] and a report with targeted metabolomics profiles in acute lymphoblastic leukemia (ALL) cell lines expressing wild type or hyperactive mutants of cN-II [23]. Concerning CD73, Ueno et al. reported on RNA sequencing of retinal cells from control and CD73-KO mice [24]. In order to continue our work on deciphering the biological roles of cN-II and CD73 in cancer cells, we submitted our panel of breast cancer cell to RNA sequencing and metabolomics. The results showed important transcriptional modifications and variations in metabolites between models in basic conditions or after exposure to adenosine and AMP.

## 2. Material and Methods

### 2.1. Cell Models and Culture

Human breast (MDA-MB-231) and lung (NCI-H292) cancer cell expressing both cN-II and CD73 were modified by CRISPR/Cas9 technology in order to have cells expressing both enzymes (cN-II^+^/CD73^+^), only CD73 (cN-II^−^/CD73^+^), only cN-II (cN-II^+^/CD73^−^) or none (cN-II^−^/CD73^−^) as explained before [20,21]. In addition, cN-II-expressing and CD73-deficient human follicular lymphoma cells (RL) were used to generate CRISPR/Cas9-mediated cN-II-deficient cells as described before [20,21] using the guideRNA sequence 5′-AACCTCTTGGTCTGTGCACA-3′. All cells were from American Type Culture Collection (ATCC) and cultivated in RPMI medium (Gibco) supplemented with 10% fetal bovine serum (Thermofischer Scientific, Waltham, MA, USA), 2 mM L-glutamine, 100 U/mL penicillin, 100 mg/mL streptomycin, and 2 µg/mL fugizone at 37 °C with 5% CO_2_. Cells were tested for *Mycoplasma* every two weeks and authenticated by sequencing.

### 2.2. RNA Sequencing

Cells were cultured in absence or presence of adenosine or AMP (1600 µM) for 1 h in two separate experiments, and harvested when reaching approximately 80% confluence. mRNA expression was analyzed by RNAsequencing (Illumina NextSeq 500) at the ProfileXpert platform (UCBL, Lyon, France) after culture in classical conditions. RNA was extracted using RNeasy mini kit (Qiagen, Les Ulis, France). Quality of samples was verified by Bioanalyzer 2100 (Agilent) and RNA was quantified by Nanodrop. After, 5 µg of RNA was enriched by NextFlex Rapid Directional mRNA-Seq kit (Bioo-Sientific, Austin, TX, USA). Quality of samples were again verified by Bioanalyzer 2100 and quantified by qPCR. Samples were put on Flow Cell High Output. Amplification and sequencing were performed with Illumina NextSeq 500 using Single Reads of 75 bp. All RNAseq samples passed quality control in terms of number of reads per sample and read quality. Trimming of single reads was performed using cutadapt v1.9.1 software. Then, the reads were mapped using Tophat2 (v2.1.1) software with default parameters on the human genome GRCh37. Alignment rates to the human genome were above 92.3%. The fragments per kilobases of exon per million mapped reads (FPKM) values were then computed for each gene using Cufflinks (v2.1.1) software as described earlier [25], and compared to cN-II^+^/CD73^+^ cells with a cutoff at 5-fold difference and *p* < 0.05 with Student’s *t*-test for positive samples as defined by FPKm > 1. Pathway analysis was performed with a cut-off at 2-fold difference and *p* < 0.05 using Database for annotation, visualization, and integrated discovery (DAVID) tools, version 6.8.

### 2.3. Quantitative RT-PCR

Gene expression was determined by quantitative RT-PCR as described in detail before [20]. Briefly, mRNA was extracted with Qiagen column extraction kit and reverse transcribed with M-MLV reverse transcriptase (InVitrogen, Cergy Pontoise, France). RT-PCR reactions were done in 5 µL with Takyon NO ROX SYBR Mastermix blue dTTP (Eurogentec, Angers, France) on a Lightcycler (LC480, Roche Life Science, Penzberg, Germany). Quantification was done with the ΔΔCT method with 28S RNA for normalization. Primers used for each gene are indicated in Supplementary Table S1.

### 2.4. Targeted Metabolomics

Cells were seeded in T75 flasks and incubated for 72 h with replacement of media after 48 h. Adenosine and AMP (1600 µM) were added when reaching approximately 80% confluence, and 1 h before metabolite extraction. Cells were washed twice with cold PBS in the flask and 3 mL of cold MeOH/H_2_O/HCOOH 68.5/28.5/3 were added. After 5 min incubation on ice, the supernatant was recovered and aliquoted in 1.5 mL tubes that were frozen for 20 s in liquid nitrogen and stored at −80 °C until analysis.

Before analysis, a mix of 24 internal standards was added to cell extracts. After centrifugation, the supernatant was evaporated under nitrogen and the dry residue was resuspended in 200 µL mobile phase. Liquid chromatographic (LC) separation was performed on an Ultimate 3000 system (ThermoFisher Scientific™, Bremen, Germany), using a Synergi HydroRP column (250 × 2; 4 µm, Phenomenex) 0.1% of formic acid in water and methanol as mobile phases. The LC system was coupled with a Q-Exactive Plus Orbitrap mass spectrometer (ThermoFisher Scientific™, Bremen, Germany) equipped with a heated electrospray ionization source operated in positive and negative mode. Metabolites were identified using retention time, exact mass and isotopic pattern of the molecular ion, exact mass of the fragments and comparison of the fragmentation spectrum with a homemade database.

## 3. Results

### 3.1. Gene Expression in 5′-Nucleotidase-Deficient Cancer Cells

**Figure 1:**
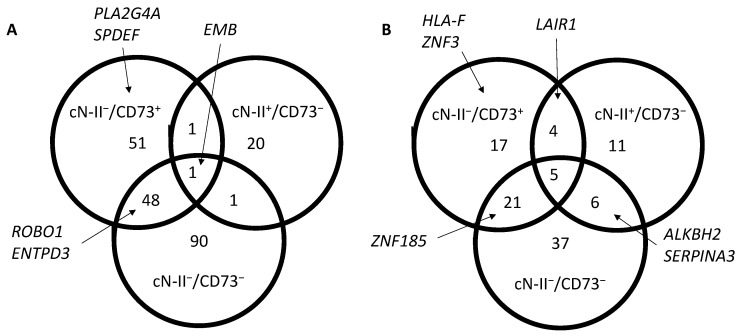
Venn diagrams showing the number of transcripts with at least a 5-fold higher (**A**) or lower (**B**) expression than in cN-II^+^/CD73^+^ cells, and with a *p*-value lower than 0.05. Only transcripts that were positive (FPKm > 1) in at least one model were included in the analysis. Complete list of transcripts is available in Supplementary Table S2.

**Figure 2:**
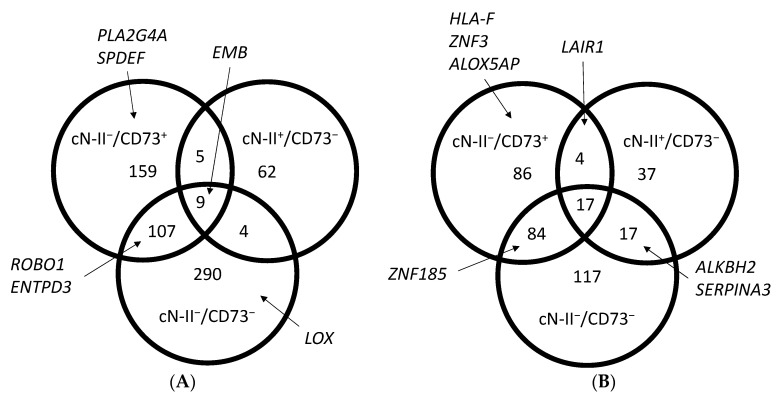
Venn diagrams showing the number of transcripts with at least a 2-fold higher (**A**) or lower (**B**) expression than in cN-II^+^/CD73^+^ cells, and with a *p*-value lower than 0.05. Only transcripts that were positive (FPKm > 1) in at least one model were included in the analysis. Complete list of transcripts is available in Supplementary Table S2.

We studied the transcriptome in our four MDA-MB-231 models cultured in standard conditions and compared expression levels to cN-II^+^/CD73^+^ cells. A total of 212 transcripts were at least 5-fold upregulated, with 48 in the two cN-II-deficient models, 1 (*CAP1*) in the two CD73-deficient models, and 1 (*EMB*) in all three models (Figure 1A and Supplementary Table S2). On the other hand, a total of 101 genes were at least 5-fold downregulated with 21 in common for the two cN-II-deficient models, 6 in the two CD73-deficient models, and 5 (*C3*, *GRAMD1B*, *POU2F2*, *SAA1*, and *TNS1*) in all three models (Figure 1B). When looking at 2-fold changes, 636 transcripts were upregulated and 362 were downregulated (Figure 2).

In order to confirm the RNA sequencing data, we selected 13 genes that were variously modified in the different models, and evaluated their expression by quantitative RT-PCR. For eight genes, the results from RNA sequencing were completely or partially confirmed (Table 1). This was in particular true for genes overexpressed in cN-II-deficient cells (*EMB*, *ENTPD3*, *LOX*, *PLA2G4A*, *ROBO1*, and *SPDEF*), but also for *LAIR1* and *SERPINA3* that were confirmed to be downregulated in cN-II^+^/CD73^−^ cells. We used other 5′-nucleotidase-deficient cell model to evaluate the cell- or tissue-specificity of the observed modifications (Table 2). For cN-II-deficient RL cells (Supplementary Figure S1), we did not see any modification in the expression of the 13 genes, whereas in NCI-H292 models, we observed a slight increase of *EMB* (1.7–1.8-fold) in all models, an increase of *ENTPD3* (1.9-fold) and *LOX* (2-fold) in cN-II^−^/CD73^+^ cells, a 2.4-fold decrease of *ROBO1* in CD73-deficient cells, and a 4.1-fold increase for *SPDEF* in cN-II^−^/CD73^−^ cells. Except for the expression of *ROBO1*, these modifications were similar as for MDA-MB-231 cells suggesting that some of the mechanisms and interplays involved in these modulations might be shared between solid tumors but not with hematological malignances.

### 3.2. Pathway-Directed Analysis of Gene Expression Profiles

A pathway-directed analysis identified several pathways as modified when each cell line was compared to cN-II^+^/CD73^+^ cells (Table 3 and Supplementary Table S3). Based on the activity of the proteins in nucleotide metabolism [26,27,28] and on previous results published concerning their involvement in migration [20,21] and energy metabolism [9,10,19,29,30], we find it interesting to see modifications in nicotinate and nicotinamide metabolism for cN-II^+^/CD73^−^ cells, in ECM-receptor interaction and cell adhesion molecules for all cN-II deficient cells, and for glucagon signaling pathway for cN-II^−^/CD73^−^ cells.

### 3.3. Transcriptional Regulation by Adenosine and AMP

**Figure 3:**
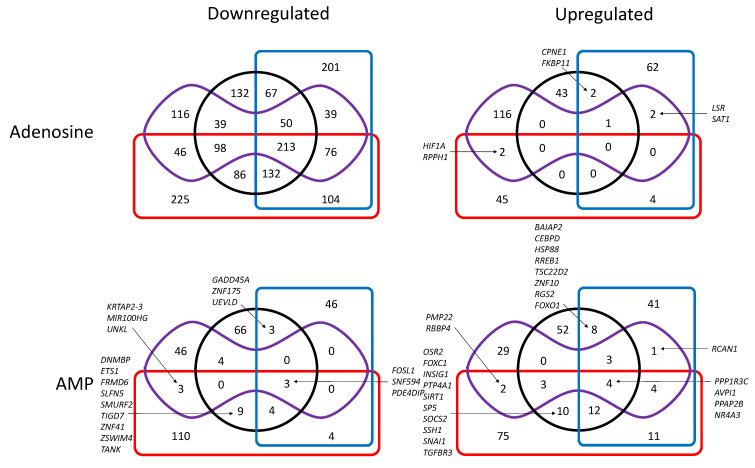
Venn diagram showing the number of at least 2-fold down- (**left**) or upregulated (**right**) transcripts (*p* < 0.05) in cells exposed to adenosine (upper panel) or AMP (lower panel) as compared to unexposed cells. Black circle: cN-II^+^/CD73^+^; red rectangle: cN-II^−^/CD73^+^; blue rectangle: cN-II^+^/CD73^−^; purple shape: cN-II^−^/CD73^−^. When the number of transcripts is less than 10 in given groups, the names of transcripts are indicated. Complete list of transcripts is available in Supplementary Tables S4 and S5.

**Figure 4:**
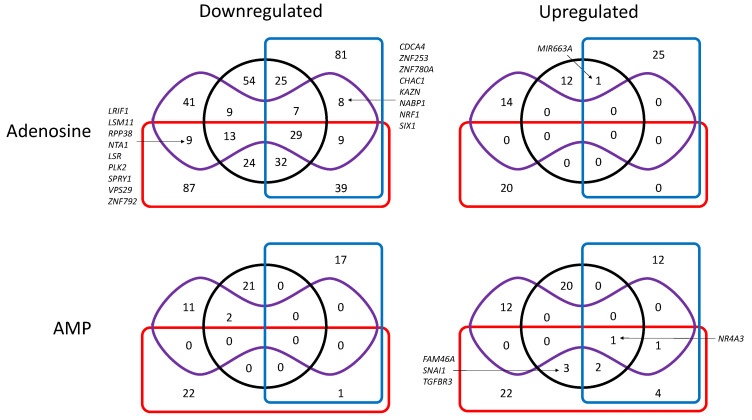
Venn diagram showing the number of at least 5-fold down- (**left)** or upregulated (**right**) transcripts (*p* < 0.05) in cells exposed to adenosine (upper panel) or AMP (lower panel) as compared to unexposed cells. Black circle: cN-II^+^/CD73^+^; red rectangle: cN-II^−^/CD73^+^; blue rectangle: cN-II^+^/CD73^−^; purple shape: cN-II^−^/CD73^−^. When the number of transcripts is less than 10 in given groups, the names of transcripts are indicated. Complete list of transcripts is available in Supplementary Tables S4 and S5.

We also studied the transcriptional regulation in our MDA-MB-231 cell models after exposure to high concentrations of adenosine or AMP for 1 h (Figure 3 and Figure 4, Supplementary Tables S4 and S5). First, it was evident that adenosine has a negative effect on transcription in our experimental conditions, with 1624 and 467 transcripts being at least 2-fold or 5-fold decreased (*p* < 0.05) respectively in at least one cell model after exposure to adenosine. These values were only 205 and 72, respectively, when considering upregulated transcripts after exposure to adenosine. As many as 213 transcripts were decreased >2-fold in all cell models exposed to adenosine, whereas 67 decreased only in cells expressing cN-II, 86 in cells expressing CD73, 39 in CD73-deficient cells, and 46 in cN-II-deficient cells (Figure 3). When looking at transcripts with at least a 5-fold decrease, there were still 29 downregulated in all four models, whereas 25 decreased only in cells expressing cN-II, 24 in cells expressing CD73, 8 (*CDCA4*, *ZNF253*, *ZND780A*, *CHAC1*, *KAZN*, *NABP1*, *NRF1*, and *SIX1*) were downregulated only in CD73-deficient cells and 9 (*LRIF1*, *LSM11*, *RPP38*, *NTA1*, *LSR*, *PLK2*, *SPRY1*, *CPS29*, *ZNF792*) in cN-II-deficient cells (Figure 4).

For cells exposed to AMP, there was, as expected, a much higher effect on CD73-proficient cells than on corresponding CD73-deficient cells (Figure 3 and Figure 4), suggesting an impact of CD73-dependant AMP metabolites, rather than a direct impact of this monophosphorylated nucleotide. Indeed, a total of 435 2-fold modifications (up- or downregulations) were observed in cN-II^+^/CD73^+^ and cN-II^−^/CD73^+^ cells (corresponding to 392 different genes, and 319 genes only modified in these cells) as compared to 246 modifications in cN-II^+^/CD73^−^ and cN-II^−^/CD73^−^ cells (233 genes in total and 163 only in these cells). For transcripts with at least 5-fold modifications, we observed one increase in all four models (*NR4A3*), and three increases (*FAM46A*, *SNAI1*, and *TGFBR3*) in CD73-expressing cells.

### 3.4. Metabolic Modifications in Cell Models

Our metabolomics approach allowed us to quantify 73 intracellular metabolites in the four cell models in basal conditions and exposed to adenosine or AMP (Supplementary Table S6). When comparing unexposed cell models (with *p* < 0.05), we observed increases of glutamic acid (1.4-fold), alanine (1.7-fold), O-propanoylcarnitine (1.7-fold), and CMP (1.7-fold) in cN-II^−^/CD73^+^ cells, of acetylcarnitine (2.9-fold), and N-acetylputrescine (2.5-fold) in cN-II^+^/CD73^−^ cells, and of glutamic acid (1.7-fold), CMP (2.4-fold), and IMP (4.7-fold) in cN-II^+^/CD73^−^ cells. In addition, trends to differences were seen for several other metabolites as can be seen on Supplementary Figure S2A. Exposure to adenosine induced many important modifications in cN-II^+^/CD73^+^ cells, as for example the expected increase in purine metabolites such as adenosine (95-fold), inosine (36-fold), hypoxanthine (8.4-fold) and xanthine (2.1-fold), but also for many non-nucleotide metabolites (Supplementary Figure S2B). Overall, cN-II- and/or CD73-deficient cells responded to a lesser extent to exposure to adenosine, but showed all increases in intracellular adenosine (50–76-fold) and inosine (11–42-fold). When exposed to AMP, cN-II^+^/CD73^+^-cells had much less modifications as compared to the exposure to adenosine (Supplementary Figure S2C). We did see however increases in adenosine (7.2-fold), inosine (14-fold), hypoxanthine (3.8-fold), and xanthine (1.3-fold). More importantly, CD73-deficient cells did not accumulate adenosine and its catabolites, but we did see an increase in AMP. Different hypotheses can explain this observation. First, AMP can be degraded by other extracellular nucleotidases than CD73 [31] and enter the cell as adenosine. This dephosphorylation is expected to be less efficient than with CD73, and intracellular adenosine being rather phosphorylated to AMP by adenosine kinase as compared to deaminated to inosine when at high concentration [32]. Second, if these cells lack the possibility to dephosphorylate AMP in the extracellular compartment, we expect this either to be a contamination by extracellular AMP or the result of an unexpected entry of AMP into cells.

We further combined the analysis of intracellular metabolites with results from RNAseq on gene products surrounding studied metabolites. Here, we used a much more conservative analysis with at least 1.1-fold modifications and *p* < 0.10, and identified a total of 57 different genes in various conditions (Supplementary Table S7). The modifications we observed in the different conditions are included in Supplementary Figure S2A–C. Out of the 34 different genes identified in unexposed cells, we selected 12 for validation by quantitative RT-PCR (Table 4). We validated the 1.5–2.0-fold increases of *CPS1* in cN-II-deficient cells and of *PDHA1* in cN-II^+^/CD73^−^ cells, but these were not found in NCI-H292 or RL cell models, again suggesting tissue- or cell-specific behaviors.

## 4. Discussion

Multi-omics approaches have gained interest in biology research, allowing analyzing in a non-targeted way various cellular parameters simultaneously such as genomic sequences, mRNA expression levels, protein expression levels, and metabolite occurrence. We have been interested in the roles of two 5′-nucleotidases in cellular biology. CD73 and cN-II have clearly described activities in nucleotide metabolism, but both our work and the work of others suggests they are involved in the regulation of cellular behaviors that are not obviously related to nucleotide metabolism. Therefore, we analyzed both mRNA expression levels and metabolites in our already published cell models [20,21].

Our results show that the cell models have a large number of transcriptional modifications, some of which are specific to one protein or the other, and some are found in both cN-II- and CD73-deficient models. The identification of modifications in the focal adhesion pathway in cN-II-negative cells and in cell adhesion molecules in cN-II^−^/CD73^−^ cells is of particular interest, given the already published evidence of cN-II in migration in this cell model [20]. This opens up for further investigations about the role of cN-II in the cellular interplay with the microenvironment and surrounding cells, using multicellular 3D models or relevant in vivo models. In a study on schizophrenia, Duarte R.R.R. et al. performed siRNA-mediated cN-II knockdown in human neuronal progenitor cells and showed decreases in genes within pathways of ribosomes, nonsense-mediated mRNA decay, protein complex disassembly, and cytoskeleton constituents [22]. Six genes reported as being modified in this experiment were found up- or downregulated as well in our MDA-MB-231 cells without cN-II. These are *AHNAK2*, *DSTN*, *GTF2I*, *NSUN5*, *PLCE1*, and *SLC15A4*. However, the fold-change and whether they are up- or downregulated were not always the same, indicating tissue- or cell-specificity. Additionally, there is a fundamental difference between the two experimental set-ups as our cells have the possibility to adapt to the 5′-nucleotidase deficiency, whereas siRNA-mediated knockdown is both partial and short-termed.

Including the conditions with exposure to adenosine and AMP allowed us to observe the impressive transcriptional inhibition exerted by adenosine in this cell model. It is however difficult to conclude on whether this is due to interaction with eventual membrane receptors or to the uptake of adenosine through nucleoside transporters [20].

Using targeted metabolomics, we were able to detect and quantify 73 intracellular metabolites. Of main interest and related to purine metabolism, we observed a 4.7-fold increase in IMP in cN-II^−^/CD73^−^ cells and huge variations in purine metabolites in cells exposed to adenosine or, to a lesser extent, AMP. The concentration used for these incubations has been shown to decrease cell proliferation and induce cell death [20], suggesting that the transcriptional and metabolic modifications observed might be involved in this. However, the short incubation time used in the current study allowed us to avoid these issues. We used formic acid for cell lysis before metabolite analysis. This might be associated with a partial degradation of nucleotides into nucleosides, inducing a potential technical bias. However, this is expected to be similar in all experimental conditions and therefore not influence the comparisons between cell models.

Moriyama et al. reported on metabolites in two different cell lines for acute lymphoblastic leukemia (Nalm6 and REH) expressing either wild type or a hyperactive mutant (R238W) of cN-II [23]. Important differences were observed between wild-type expressing and mutant-expressing cells when exposed to 6-mercaptopurine, but unexposed cells had only minor differences in metabolites, of which several were in purine metabolism. This is in line with earlier hypotheses about cN-II playing minor roles in the cells at basal conditions, but being important during stress. It should be noted that we used knock-out cell models whereas Moriyama et al. used cells expressing the protein but with a higher activity. It has already been shown that cN-II mediates roles through protein–protein interaction that might be independent of its activity [33]. A high number of potential cN-II and CD73 interactors appear in public databases, and these could constitute a starting point for further investigations on functional interactions and regulations by these two proteins.

Overall, we used a double-sided methodology to get pictures of transcriptional and metabolic differences between our models with or without cN-II and/or CD73 expression. Further experiments on these and other models will enable confirmation of the variations as well as the functional implication of the two proteins in the observed differences. Additionally, transcriptional differences reported here could constitute the basis for non-cancer related research, with confirmation of similar regulations in other settings suggested in Table 3 for example (coagulation, infections, digestion…). Finally, the quantification of extracellular metabolites, and in particular nucleosides (adenosine) and nucleobases (hypoxanthine), would be informative as well in order to conclude on the pathophysiological roles of cN-II and CD73.

## Figures and Tables

**Table 1:** Validation of RNA sequencing results by quantitative RT-PCR in unexposed cells. Values for qRT-PCR are means of four different samples ± standard deviation. ^a^: *p* < 0.05 for comparison to cN-II^+^/CD73^+^ cells as determined by Student’s *t*-test on FKPM values from RNA sequencing or by one-way ANOVA test for quantitative RT-PCR results. Grey lines indicate completely or partially similar results between RNA sequencing and qRT-PCR.

	RNA Sequencing				qRT-PCR			
Gene	cN-II^+^/CD73^+^	cN-II^−^/ CD73^+^	cN-II^+^/ CD73^−^	cN-II^−^/ CD73^−^	cN-II^+^/ CD73^+^	cN-II^−^/ CD73^+^	cN-II^+^/ CD73^−^	cN-II^−^/ CD73^−^
*ALKBH2*	1.1	0.2	0.0003 ^a^	0.001 ^a^	0.8 ± 0.2	1.09 ± 0.05 ^a^	0.66 ± 0.04	1.4 ± 0.2 ^a^
*ALOX5AP*	3.2	0.7 ^a^	0.7	0.2	0.6 ± 0.3	0.7 ± 0.2	0.23 ± 0.01	0.8 ± 0.5
*EMB*	0.4	10.8 ^a^	2.5 ^a^	14.6 ^a^	0.7 ± 0.2	16.1 ± 3.9 ^a^	2.9 ± 0.7	22.2 ± 8.6 ^a^
*ENTPD3*	0.4	2.1 ^a^	0.4	4.4 ^a^	0.8 ± 0.2	3.8 ± 1.1 ^a^	1.2 ± 0.4	13.9 ± 2.3 ^a^
*HLA-F*	1.3	0.1 ^a^	0.8	0.4	0.7 ± 0.2	0.6 ± 0.2	0.55 ± 0.08	0.6 ± 0.3
*LAIR1*	1.4	0.0003 ^a^	0.08 ^a^	0.2	0.8 ± 0.2	1.1 ± 0.2 ^a^	0.30 ± 0.09 ^a^	0.83 ± 0.08
*LOX*	5.6	9.5	9.5	12.6 ^a^	0.8 ± 0.1	2.3 ± 0.7 ^a^	2.0 ± 0.3 ^a^	4.1 ± 0.7 ^a^
*PLA2G4A*	0.02	1.5 ^a^	0.02	1.6 ^a^	1.1 ± 0.4	79 ± 10 ^a^	3.1 ± 1.2	111 ± 10 ^a^
*ROBO1*	n.d.	1.6 ^a^	n.d.	2.0 ^a^	1.0 ± 0.2	90 ± 16 ^a^	5.2 ± 2.2	225 ± 17 ^a^
*SERPINA3*	2.5	0.8 ^a^	0.2 ^a^	0.1 ^a^	0.6 ± 0.3	0.6 ± 0.2	0.17 ± 0.04 ^a^	0.3 ± 0.2
*SPDEF*	0.1	15.0 ^a^	0.6	18.2	0.9 ± 0.3	14.5 ± 0.7 ^a^	2.0 ± 0.6	17.7 ± 2.2 ^a^
*ZNF185*	1.6	0.1 ^a^	0.9	n.d.^a^	0.9 ± 0.1	1.3 ± 0.2 ^a^	1.30 ± 0.03 ^a^	0.82 ± 0.07
*ZNF3*	1.4	0.1 ^a^	0.009	0.5	0.8 ± 0.1	0.8 ± 0.1	0.9 ± 0.1	0.8 ± 0.1

**Table 2:** Expression of certain studied genes in other cell models as determined by quantitative RT-PCR in unexposed cells. Values are means of four different samples ± standard deviation. ^a^: *p* < 0.05 for comparison to cN-II^+^/CD73^+^ cells as determined by one-way ANOVA test. n.d.: not detected.

	NCI-H292				RL	
Gene	cN-II^+^/ CD73^+^	cN-II^−^/ CD73^+^	cN-II^+^/ CD73^−^	cN-II^−^/ CD73^−^	cN-II^+^	cN-II^−^
*ALOX5AP*	0.6 ± 0.4	0.4 ± 0.2	0.6 ± 0.1	0.5 ± 0.3	0.9 ± 0.2	1.2 ± 0.3
*EMB*	1.4 ± 0.5	2.5 ± 0.7 ^a^	2.4 ± 0.1 ^a^	2.5 ± 0.5 ^a^	1.0 ± 0.2	1.4 ± 0.1
*ENTPD3*	0.8 ± 0.2	1.5 ± 0.5 ^a^	1.2 ± 0.3	1.3 ± 0.5	n.d.	n.d.
*LAIR1*	n.d.	n.d.	n.d.	n.d.	1.5 ± 0.7	0.8 ± 0.9
*LOX*	1.6 ± 0.8	3.17 ± 0.06 ^a^	2.6 ± 0.3	2.7 ± 1.2	0.9 ± 0.4	0.8 ± 0.4
*PLA2G4*	1.4 ± 0.3	1.3 ± 0.2	1.4 ± 1.0	1.4 ± 0.5	0.7 ± 0.6	8.0 ± 8.2
*ROBO1*	1.7 ± 1.2	2.9 ± 0.7	0.7 ± 0.2 ^a^	0.7 ± 0.2 ^a^	1.2 ± 0.2	0.8 ± 0.1
*SERPINA3*	n.d.	n.d.	n.d.	n.d.	n.d.	n.d.
*SPDEF*	0.8 ± 0.4	1.4 ± 0.3	1.7 ± 0.7	3.3 ± 2.3 ^a^	n.d.	n.d.

**Table 3:** Conservative pathway analysis of RNA sequencing data. The table indicates pathways with *p* < 0.1 with a cutoff of 2-fold change in expression levels. In bold: pathways detailed in Supplementary Table S3.

Model	Pathway Term	Genes	*p*-Value
cN-II^+^/CD73^−^	Pantothenate and CoA biosynthesis	4	0.00053
	HTLV-I infection	6	0.0083
	Herpes simplex infection	8	0.025
	**Nicotinate and nicotinamide metabolism**	3	0.028
	Intestinal immune network for IgA production	3	0.068
	Tuberculosis	5	0.077
cN-II^−^/CD73^+^	**ECM-receptor interaction**	8	0.0065
	Hippo signaling pathway	10	0.015
	Protein digestion and absorption	7	0.024
	Complement and coagulation cascades	6	0.031
	**Pathways in cancer**	17	0.042
	Mucin type O-Glycan biosynthesis	4	0.043
	Ras signaling pathway	11	0.062
	**Focal adhesion**	10	0.080
	Rap1 signaling pathway	10	0.087
cN-II^−^/CD73^−^	Complement and coagulation cascades	8	0.0065
	**Cell adhesion molecules**	11	0.016
	Oxytocin signaling pathway	11	0.023
	Proteoglycans in cancer	13	0.028
	*Staphylococcus aureus* infection	6	0.029
	Viral myocarditis	6	0.036
	Rap1 signaling pathway	13	0.039
	**Glucagon signaling pathway**	8	0.040
	Protein digestion and absorption	7	0.064
	Malaria	5	0.072
	Inflammatory mediator regulation of TRP channels	7	0.096
	Epstein–Barr virus infection	8	0.099

**Table 4:** Quantitative RT-PCR on identified genes in unexposed cells of the metabolic study. Values are means of four different samples ± standard deviation. ^a^: *p* < 0.05 for comparison to cN-II^+^/CD73^+^ cells as determined by one-way ANOVA test. ^b^: *p* < 0.05 for comparison to cN-II^+^/CD73^−^ cells as determined by one-way ANOVA test.

	MDA-MB-231				NCI-H292				RL	
Gene	cN-II^+^/ CD73^+^	cN-II^−^/ CD73^+^	cN-II^+^/ CD73^−^	cN-II^−^/ CD73^−^	cN-II^+^/ CD73^+^	cN-II^−^/ CD73^+^	cN-II^+^/ CD73^−^	cN-II^−^/ CD73^−^	cN-II^+^	cN-II^−^
*ACO1*	1.1 ± 0.7	0.9 ± 0.3	0.9 ± 0.6	1.13 ± 0.09	1.2 ± 1.3	1.7 ± 0.8	0.7 ± 0.2	0.8 ± 0.2	2.5 ± 1.5	5.3 ± 0.3
*ASS1*	1.5 ± 0.6	2.0 ± 1.8	2.5 ± 1.0	2.0 ± 0.2	1.0 ± 0.4	1.0 ± 0.6	0.8 ± 0.2	0.8 ± 0.2	0.7 ± 0.3	3.0 ± 1.0
*BCAT1*	0.8 ± 0.2	1.1 ± 0.5	0.667 ± 0.002	0.83 ± 0.03	1.5 ± 0.7	1.5 ± 0.3	0.9 ± 0.4	0.8 ± 0.4	1.1 ± 0.2	1.3 ± 0.3
*CPS1*	0.8 ± 0.4	1.7 ± 0.5 ^a^	0.7 ± 0.2	1.82 ± 0.08 ^a,b^	0.8 ± 0.4	0.7 ± 0.6	0.3 ± 0.1	0.4 ± 0.1	1.1 ± 0.2	1.4 ± 0.4
*CTH*	0.8 ± 0.2	0.5 ± 0.2	0.47 ± 0.01	0.5 ± 0.1	2.0 ± 1.2	1.7 ± 0.6	1.6 ± 0.5	1.5 ± 0.5	1.5 ± 0.7	1.8 ± 0.4
*ECHS1*	0.7 ± 0.4	0.9 ± 0.5	0.66 ± 0.04	1.2 ± 0.3	1.1 ± 0.3	1.2 ± 0.5	1.0 ± 0.5	0.9 ± 0.4	1.0 ± 0.2	1.3 ± 0.3
*FH*	0.6 ± 0.5	0.6 ± 0.3	0.567 ± 0.004	0.7 ± 0.2	1.5 ± 0.7	1.6 ± 0.6	1.3 ± 0.5	1.1 ± 0.6	0.8 ± 0.2	1.0 ± 0.2
*GLUD1*	0.9 ± 0.3	1.2 ± 0.4	0.1 ± 0.2	1.13 ± 0.08	1.2 ± 0.6	1.5 ± 1.3	0.9 ± 0.2	0.9 ± 0.3	1.1 ± 0.3	1.5 ± 0.3
*GNPNAT1*	0.8 ± 0.2	0.8 ± 0.4	0.9 ± 0.4	0.9 ± 0.2	0.9 ± 0.3	2.0 ± 2.0	1.8 ± 0.5	1.2 ± 0.2	1.0 ± 0.3	1.7 ± 0.3
*IVD*	0.7 ± 0.4	0.7 ± 0.3	0.70 ± 0.06	0.98 ± 0.06	1.1 ± 0.4	1.1 ± 0.5	0.7 ± 0.3	0.8 ± 0.3	1.4 ± 0.2	2.5 ± 0.3
*PDHA1*	0.94 ± 0.09	1.0 ± 0.4	1.7 ± 0.3 ^a^	1.15 ± 0.09	1.4 ± 0.6	1.3 ± 0.4	1.0 ± 0.3	1.0 ± 0.4	1.0 ± 0.2	1.2 ± 0.2
*TPI1*	2.7 ± 2.4	2.1 ± 1.1	1.1 ± 0.2	1.4 ± 0.2	1.9 ± 1.0	2.0 ± 0.6	1.3 ± 0.3	1.5 ± 0.7	1.2 ± 0.2	1.5 ± 0.4

## Data Availability

All raw data and models can be obtained by reasonable request to L.P.J.

## Supplementary Materials

The following are available online at https://www.mdpi.com/article/10.3390/cells10112918/s1, Figure S1: Expression of cN-II in CRISPR/Cas9-transfected RL cells. Cont- and Cl2-cells were used in the experiments. Cont: control sequence; Cl1: Clone 1 with cN-II-targeting sequence; Cl2: Clone 2 with cN-II-targeting sequence; Figure S2A–C: Comparison of metabolites and transcripts between modified cell lines and cN-II+/CD73+ cells (A) or between unexposed cells and cells exposed to adenosine (B) or AMP (C). Increases in metabolites and genes are indicated in green and decreases in red. Values indicate fold-changes for metabolites. Filled markers indicate *p* < 0.05 and only surrounded markers indicate 0.05 < *p* < 0.10 for metabolites. Cut-off for RNA sequencing data was *p* < 0.10 and 1.1-fold modification. ♦: cN-II^+^/CD73^+^, ■: cN-II^+^/CD73^−^, ▲: cN-II^−^/CD73^+^, ⬤: cN-II^−^/CD73^−^. Metabolites with changes in some conditions that do not appear on the figures are creatine, aminoisoburyrate, N-acetylspermidine, O-propanoylcarnitine, proline, uridine and homocysteine. Details for metabolic modifications and RNA sequencing results are in Supplementary Tables S2, S4–S7; Table S1: Primers used for quantitative RT-PCR. ACO1: aconitase 1; ALKBH2: DNA oxidative demethylase; ALOX5AP: arachidonate 5-lipoxygenase activating protein; ASS1: argininosuccinate synthase 1; BCAT1: branced chain amino acid transaminase 1; CPS1: carbamoyl-phosphate synthase 1; CTH: cystathionine gamma-lyase; ECHS1: enoyl-CoA hydratase short-chain 1; EMB: embigin; ENTPD3: ectonucleoside triphosphate diphosphohydrolase 3; FH: fumarate hydratase; GLUD1: glutamate dehydrogenase 1; GNPNAT1: glucosamine-phosphate N-acetyltransferase 1; HLA-F: major histocompatibility complex class I F; IVD: isovaleryl-CoA dehydrogenase; LAIR1: leukocyte associated immunoglobulin like receptor 1; LOX: lysyl oxidase; PDHA1: pyruvate dehydrogenase E1 subunit alpha 1; PLA2G4A: phospholipase A2 group IVA; ROBO1: roundabout guidance receptor 1; SERPINA3: serpin family A member 3; SPDEF: SAM pointed domain containing ETS transcription factor; TPI1: triosephosphate isomerase 1; ZNF185: zinc finger protein 185 with LIM domain; ZNF3: zinc finger protein 3; Table S2: List of genes modified at least 2-fold in modified cell models as compared to cN-II^+^/CD73^+^ cells. There is one sheet for each cell line. In red, genes with higher expression in modified cells as compared to control cells. In green, genes with higher expression in control cells as compared to modified cells; Table S3: Genes with modifications in selected pathways from Table 3. In bold: genes with multiple appearances in the selected pathways; Table S4: List of genes modified at least 2-fold in the four cell models after exposure to adenosine. There is one sheet for each cell line. In red, genes with higher expression in unexposed cells. In green, genes with higher expression in exposed cells; Table S5: List of genes modified at least 2-fold in the four cell models after exposure to AMP. There is one sheet for each cell line. In red, genes with higher expression in unexposed cells. In green, genes with higher expression in exposed cells; Table S6: List of the 73 studied metabolites in the four cell models exposed or not to adenosine and AMP. Values are means and standard deviation from three or four independent experiments. Colored cells indicate increased (green) or decreased (red) values with *p* < 0.05 or *p* < 0.10 when only surrounded; Table S7. List of genes around studied metabolites with at least 1.1-fold modifications in expression levels and with *p* < 0.10. There is one sheet for each cell line, with comparisons to cN-II+/CD73+ cells and to cells not exposed to adenosine or AMP.
