# Supplementary material for: Transcriptional and Metabolic Investigation in 5′-Nucleotidase Deficient Cancer Cell Lines
Source: Cells. 2021 Oct 28;10(11):2918. doi: 10.3390/cells10112918 (PMC8616413; doi:10.3390/cells10112918)

Supplementary Table S1. Primers used for quantitative RT-PCR. ACO1: aconitase 1; ALKBH2: DNA oxidative demethylase; ALOX5AP: arachidonate 5-lipoxygenase activating protein; ASS1: argininosuccinate synthase 1; BCAT1: branced chain amino acid transaminase 1; CPS1: carbamoyl-phosphate synthase 1; CTH: cystathionine gamma-lyase; ECHS1: enoyl-CoA hydratase short-chain 1; EMB: embigin; ENTPD3: ectonucleoside triphosphate diphosphohydrolase 3; FH: fumarate hydratase; GLUD1: glutamate dehydrogenase 1; GNPNAT1: glucosamine-phosphate N-acetyltransferase 1; HLA-F: major histocompatibility complex class I F; IVD: isovaleryl-CoA dehydrogenase; LAIR1 : leukocyte associated immunoglobulin like receptor 1; LOX : lysyl oxidase; PDHA1 : pyruvate dehydrogenase E1 subunit alpha 1; PLA2G4A : phospholipase A2 group IVA; ROBO1 : roundabout guidance receptor 1; SERPINA3 : serpin family A member 3; SPDEF : SAM pointed domain containing ETS transcription factor; TPI1 : triosephosphate isomerase 1; ZNF185 : zinc finger protein 185 with LIM domain ; ZNF3 : zinc finger protein 3.

Supplementary Table S2. List of genes modified at least 2-fold in modified cell models as compared to cN-II$^+$/CD73$^+$ cells. There is one sheet for each cell line. In red, genes with higher expression in modified cells as compared to control cells. In green, genes with higher expression in control cells as compared to modified cells.

Supplementary Table S3. Genes with modifications in selected pathways from Table 3. In bold: genes with multiple appearances in the selected pathways.

Supplementary Table S4. List of genes modified at least 2-fold in the four cell models after exposure to adenosine. There is one sheet for each cell line. In red, genes with higher expression in unexposed cells. In green, genes with higher expression in exposed cells.

Supplementary Table S5. List of genes modified at least 2-fold in the four cell models after exposure to AMP. There is one sheet for each cell line. In red, genes with higher expression in unexposed cells. In green, genes with higher expression in exposed cells.

Supplementary Table S6. List of the 73 studied metabolites in the four cell models exposed or not to adenosine and AMP. Values are means and standard deviation from three or four independent experiments. Colored cells indicate increased (green) or decreased (red) values with $p<0.05$ or $p<0.10$ when only surrounded.

Supplementary Table S7. List of genes around studied metabolites with at least 1.1-fold modifications in expression levels and with $p<0.10$. There is one sheet for each cell line, with comparisons to cN-II+/CD73+ cells and to cells not exposed to adenosine or AMP.

Supplementary Figure S1. Expression of cN-II in CRISPR/Cas9-transfected RL cells. Cont- and Cl2-cells were used in the experiments. Cont: control sequence; Cl1: Clone 1 with cN-II-targeting sequence; Cl2: Clone 2 with cN-II-targeting sequence.

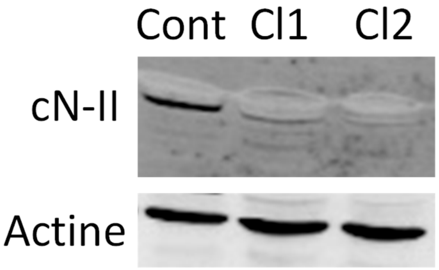

Supplementary Figure S2A-C. Comparison of metabolites and transcripts between modified cell lines and cN-II+/CD73+ cells (A) or between unexposed cells and cells exposed to adenosine (B) or AMP (C). Increases in metabolites and genes are indicated in green and decreases in red. Values indicate fold-changes for metabolites. Filled markers indicate $p<0.05$ and only surrounded markers indicate $0.05 < p < 0.10$ for metabolites. Cut-off for RNA sequencing data was $p<0.10$ and 1.1-fold modification. ◆: cN-II$^+$/CD73$^+$, ■: cN-II$^+$/CD73$^-$, ▲: cN-II$^-$/CD73$^+$, ●: cN-II$^-$/CD73$^-$. Metabolites with changes in some conditions that do not appear on the figures are creatine, aminoisoburyrate, N-acetylspermidine, O-propanoylcarnitine, proline, uridine and homocysteine. Details for metabolic modifications and RNA sequencing results are in Supplementary Tables S2, S4-S7.

Supplementary Figure S2A

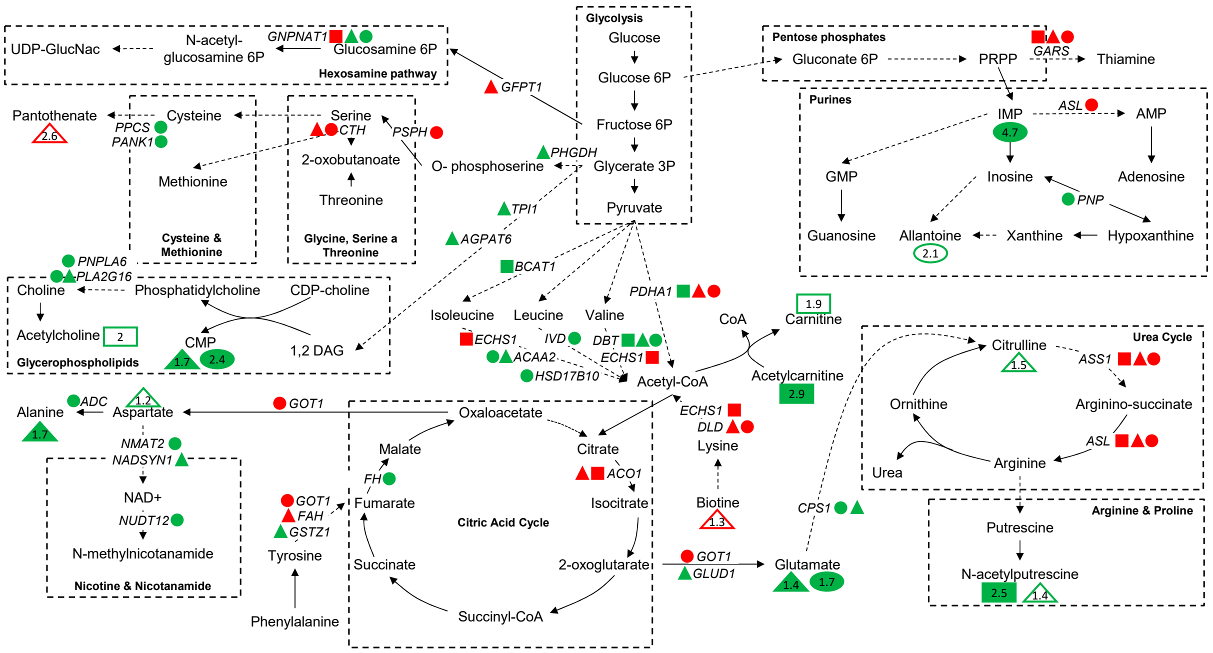

Supplementary Figure S2B

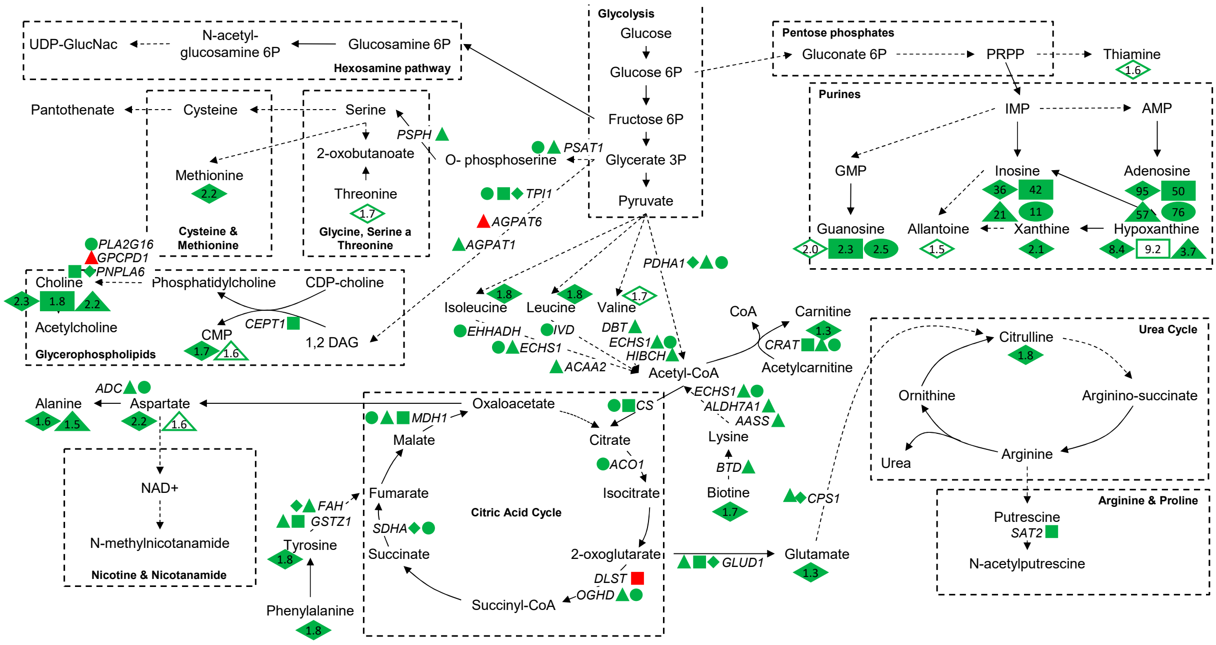

Supplementary Figure S2C

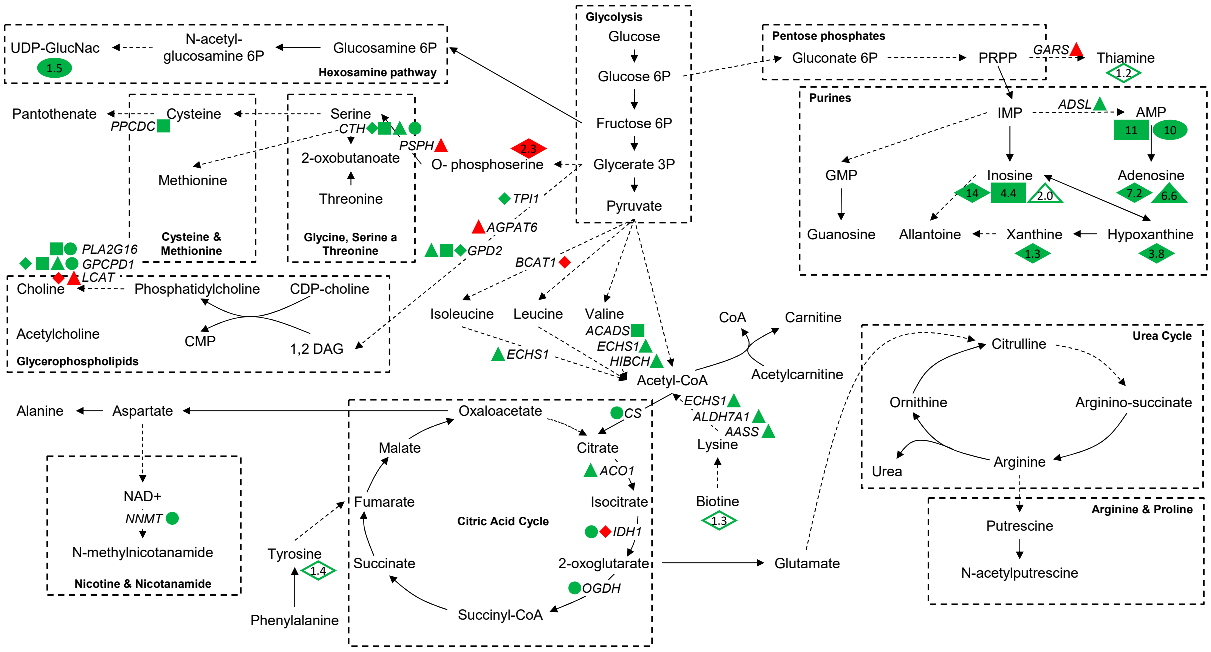

Supplement: Supplementary file 1 [file cells-10-02918-s001.zip › Supplemental material.pdf]
