# Supplementary material for: Transcriptional and Metabolic Investigation in 5′-Nucleotidase Deficient Cancer Cell Lines
Source: Cells. 2021 Oct 28;10(11):2918. doi: 10.3390/cells10112918 (PMC8616413; doi:10.3390/cells10112918)
Supplement: Supplementary file 1 [file cells-10-02918-s001.zip › Supplementary Table S1.pdf]

Supplementary Table S1. Primers used for quantitative RT-PCR. ACO1: aconitase 1; ALKBH2: DNA oxidative demethylase; ALOX5AP: arachidonate 5-lipoxygenase activating protein; ASS1: argininosuccinate synthase 1; BCAT1: branced chain amino acid transaminase 1; CPS1: carbamoyl-phosphate synthase 1; CTH: cystathionine gamma-lyase; ECHS1: enoyl-CoA hydratase short-chain 1; EMB: embigin; ENTPD3: ectonucleoside triphosphate diphosphohydrolase 3; FH: fumarate hydratase; GLUD1: glutamate dehydrogenase 1; GNPNAT1: glucosamine-phosphate N-acetyltransferase 1; HLA-F: major histocompatibility complex class I F; IVD: isovaleryl-CoA dehydrogenase; LAIR1 : leukocyte associated immunoglobulin like receptor 1; LOX : lysyl oxidase; PDHA1 : pyruvate dehydrogenase E1 subunit alpha 1; PLA2G4A : phospholipase A2 group IVA; ROBO1 : roundabout guidance receptor 1; SERPINA3 : serpin family A member 3; SPDEF : SAM pointed domain containing ETS transcription factor; TPI1 : triosephosphate isomerase 1; ZNF185 : zinc finger protein 185 with LIM domain ; ZNF3 : zinc finger protein 3.

**Gene**	**Forward primer (5’-3’)**	**Reverse primer (5’-3’)**
ACO1	CATCCTGGAGTGTGGTAGGAACA	AAGCAGCCATTCGGAATTGTGA
ALKBH2	CAACTTTGTGCTCATCAACAGG	TCTTTCATCATCTCGGTGCTC
ALOX5AP	GCAAGCTCTCACTTCCCCTTC	ATTCTGGGTCCTGCTTTCGT
ASS1	GAGCCCGAGTGGTTCACTG	CGCTATGTCCAGCAAAGGCT
BCAT1	ACGACCCTTGGGATCTGCC	CCGCAGAGTGTACCGGAGAA
CPS1	GAGGCCCATGCCACAAATCA	GTCAAGGCACAGACAGCACA
CTH	GCCAGCACTCGGGTTTTGA	AGCACTGGATGGGGCTAAGT
ECHS1	ACCCAGGTCAAGAAGCCAGT	AGTTTGCACAGCCGGAGATC
EMB	GCGAGCTGAGGGAGCAG	TCTCTGAGAGGTGGACTTGT
ENTPD3	TTTCCCTGGACACCTTCAAC	TGTATTTGGGGCCAAGTCTC
FH	AAGCTCCCTCAGCACCATGTA	CGAATGGCAAGCCAAAATTCCT
GLUD1	TCACCATGGAGCTAGCAAAA	ACAGGTGAGCGGGAGATGT
GNPNAT1	ACTGGTGGGGGAGAGTCC	TCCAGCCATTTCCCCAACAC
HLA-F	GCTGCTGTGATGTGGAGGAA	AGAGCACCCCCAGGAGAAAT
IVD	CCCGTGGACGATGCAATCAA	AAGGCCCAGGAGATCGATCG
LAIR1	CCCAGACCATCCACACGC	TCTCCAGGCGGAATGTTTGAA
LOX	GGCGACGACCCTTACAACC	GTGGACGCCTGGATGTAGTAG
PDHA1	CCTGACTTTATATGGCGATG	CGAAGCTTACAACATGGCAG
PLA2G4A	CATGCCCAGACCTACGATTT	CCCAATATGGCTACCACAGG
ROBO1	GCTAATTGACCCACGTTGCTT	CTTCCTGACGAAGACGGGAG
SERPINA3	ACTCCAGACAGACGGCTTTG	ATTCTCTCCATTCTCAACTCTGC
SPDEF	ACCTGAGTGCTGCCTCTGC	CATGCCGCTGCTGTTTGG
TPI1	GCGCTCTATATAAGTGGGCAGTGG	CGACACCGAGGTGGTTTGTG
ZNF185	GCGGATCTGAGCAACTTGTC	TTCTGGGGTACTGGGATCTG
ZNF3	CGGGAACTTGAACAGAGGCT	TGAGGAAGCACTGCCTGATT
