# Supplementary material for: Transcriptional and Metabolic Investigation in 5′-Nucleotidase Deficient Cancer Cell Lines
Source: Cells. 2021 Oct 28;10(11):2918. doi: 10.3390/cells10112918 (PMC8616413; doi:10.3390/cells10112918)
Supplement: Supplementary file 1 [file cells-10-02918-s001.zip › Supplementary Table S3.pdf]

Supplementary Table S3. Genes with modifications in selected pathways from table 3. In bold: genes with multiple appearances in the selected pathways.

**Model**	**Pathway term**	**Gene**	**Gene name**
cN-II$^+$/CD73$^-$	Nicotinate and nicotinamide metabolism	NT5C	5', 3'-nucleotidase, cytosolic
		NT5E	5'-nucleotidase ecto
		NMRK1	Nicotinamide riboside kinase 1
cN-II$^-$/CD73$^+$	ECM-receptor interaction	**COL6A2**	**Collagen type VI alpha 2 chain**
		**COL6A3**	**Collagen type VI alpha 3 chain**
		DAG1	Dystroglycan
		**FN1**	**Fibronectin 1**
		HSPG2	Heparan sulfate proteoglycan 2
		HMMR	Hyaluronan mediated motility receptor
		**ITGA2**	**Integrin subunit alpha 2**
		**LAMA3**	**Laminin subunit alpha 3**
	Pathways in cancer	CXCR4	C-X-C motif chemokine receptor 4
		MECOM	MDS1 and EVI1 complex locus
		NKX3-1	NK3 homeobox 1
		WNT7B	Wnt family member 7B
		ADCV3	Adenylate cyclase 3
		AXIN2	Axin 2
		BMP4	Bone morphogenetic protein 4
		CSF2RA	Colony stimulating factor 2 receptor alpha subunit
		FGF5	Fibroblast growth factor 5
		**FN1**	**Fibronectin 1**
		FZD4	Frizzled class receptor 4
		**ITGA2**	**Integrin subunit alpha 2**
		**LAMA3**	**Laminin subunit alpha 3**
		LPAR1	Lysophosphatidic acid receptor 1
		**PGF**	**Placental growth factor**
		**PDGFA**	**Platelet derived growth factor subunit A**
		**VEGFA**	**Vascular endothelial growth factor 1**
	Focal adhesion	RASGRF1	Ras protein specific guanine nucleotide releasing factor 1
		SHC3	SHC adaptor protein 3

		**COL6A2**	**Collagen type VI alpha 2 chain**
		**COL6A3**	**Collagen type VI alpha 3 chain**
		**FN1**	**Fibronectin 1**
		**ITGA2**	**Integrin subunit alpha 2**
		**LAMA3**	**Laminin subunit alpha 3**
		**PGF**	**Placental growth factor**
		**PDGFA**	**Platelet derived growth factor subunit A**
		**VEGFA**	**Vascular endothelial growth factor 1**
cN-II$^-$/CD73$^-$	Cell adhesion molecules	CD40	CD40 molecule
		L1CAM	L1 cell adhesion molecule
		CLDN23	Claudin 23
		ICOSLG	Inducible T-cell costimulator ligand
		ITGB2	Integrin subunit beta 2
		ICAM1	Intercellular adhesion molecule 1
		HLA-B	Major histocompatibility complex, class 1, B
		HLA-DRB5	Major histocompatibility complex, class II, DR beta 5
		NEO1	Neogenin 1
		NLGN1	Neuroligin 1
		SDC2	Syndecan 2
	Glucagon signaling pathway	CREB3L4	cAMP responsive element binding, protein 3 like 4
		CAMK2D	Calcium/calmodulin dependent protein kinase II delta
		CAMK2G	Calcium/calmodulin dependent protein kinase II gamma
		CPT1C	Carnitine palmitoyltransferase 1C
		PHKG2	Phosphorylase kinase catalytic subunit gamma 2
		PRKAA2	Protein kinase AMP-activated catalytic subunit alpha 2
		PRKAG2	Protein kinase AMP-activated non-catalytic subunit gamma 2
		PRKACB	Protein kinase cAMP-activated catalytic subunit beta
